# Shape of snack foods does not predict snack intake in a sample of preschoolers: a cross-over study

**DOI:** 10.1186/1479-5868-9-94

**Published:** 2012-08-06

**Authors:** Lauren E Boyer, Sara Laurentz, George P McCabe, Sibylle Kranz

**Affiliations:** 1 Undergraduate student, Department of Nutrition Science, College of Health and Human Sciences, Purdue University, B6 Stone Hall, 700 W. State Street, West Lafayette, IN, 47907-2059, USA; 2 Department of Statistics, College of Science, Purdue University, G171 Mathematical Sciences Building, 150 N. University Street, West Lafayette, IN, 47907-2066, USA; 3 Department of Statistics, College of Science, Purdue University, 932 Mathematical Sciences Building, 150 N. University Street, West Lafayette, IN, 47907-2067, USA; 4 Department of Nutrition Science, College of Health and Human Sciences, Purdue University, 204 Stone Hall, 700 W. State Street, West Lafayette, IN, 47907-2059, USA

**Keywords:** Preschool children, Food intake, Dietary quality, Snack shape

## Abstract

**Background:**

In the past decade, the proportion snacking has increased. Snack foods consumed are predominantly not nutritious foods. One potential venue to increase children’s diet quality is to offer healthy snack foods and we explored if shaped snack foods would lead to increased consumption.

**Methods:**

We investigated the consumption of high-fiber snacks (banana bread, pancakes, and sandwiches) served either in normal (round, square) or shaped (heart, hands, animals) form to preschoolers 2–5 years old attending a local child care center (n = 21). The 9 weeks long, prospective, cross-over intervention study was designed to expose each child repeatedly to each snack in each shape (4 times per snack). Snacks were served as morning or afternoon snack and caretakers’ reports were used to account for the child’s consumption of a meal preceding the study snack (breakfast or lunch).

**Results:**

There was no significant difference in snack consumption between the shaped and normal snacks. However, the mean energy intake from snacks was significantly greater for Caucasian children compared with Asian children. Further, Asian children consumed much less banana bread than the other two snacks. Overall, children who had not eaten breakfast or lunch prior to the morning or afternoon snack ate significantly more calories from the snacks (84.1 kcal, p-value < 0.0001).

**Conclusion:**

Findings of this study confirm previous research that the shape of the foods does not affect snack consumption in children. However, we also report two unexpected findings: a) the strong interaction between ethnicity and snack consumption and b) that Asian children consumed much less banana bread than Caucasian children. The role of children’s ethnic background profoundly affects snack preference and must be considered in the study of children’s eating behaviors and in interventions to promote healthy eating habits.

## Background

Child care centers might be critical settings for effort to improve children’s eating patterns [1,2]. In 2007, approximately 55% of children ages 3 to 6 years were enrolled in center-based care [3]. Analyses of dietary intakes of children attending full-time child care centers across the U. S. have shown that children have lower than recommended intake of whole grains, fruits, vegetables, and dairy [2,4]. On the other hand, children are consuming excessive amounts of added sugar and saturated fat. An important nutrient for children’s health that is commonly underconsumed is dietary fiber, which can be partially attributed to the low intake of high-fiber foods such as whole grains, fruits, vegetables, and legumes [5,6].

Along with the rise in obesity prevalence, the incidence of children’s snacking has increased [7,8]. From 1977 to 2006, children increased the consumption of energy from snacks by 168 to approximately 491 calories per day which is approximately 27% of their daily intake [8]. In addition, while most children consume an estimated three snacks per day, there was a decline of food consumption at the primary meals: breakfast, lunch, and dinner. The largest proportion of foods consumed as snacks were known predictors of low diet quality, such as desserts, savory snacks, and sweetened beverages [8]. For instance, between 1977–1978 and 2003–2006, the proportion of calories from snacks that were candy or salty snacks significantly increased from 5.7% to 8.5% and from 8.1% to 15.7%, respectively [8].

It is well known that children consume suboptimal levels of fruits, vegetables, whole grains, and legumes, although federal nutrition guidance has encouraged consumption of these food groups for several decades. In an effort to increase fiber consumption, the shape of food might be used to increase children’s intake, as data from product marketing towards children indicates a positive effect of shapes to create “fun foods” [9]. In marketing, foods are strategically portrayed as fun either by association or by specific characteristics of those foods. Unfortunately many of these “fun foods” are lacking nutrients and are high in calories, such as carbonated beverages, fast food, and sugary cereals. Fitzhugh and Lobstein completed an analysis of the nutritional quality of foods targeted specifically at children in the United Kingdom [10]. Of 358 food products considered to be marketed toward children, they found that 77% contained high levels of sugar, salt, saturated fat or total fat. Interestingly, these products were often offered in child-oriented product shapes like animals or familiar cartoon characters. In addition, in Canada 89% of 367 products targeted toward children, including foods in child-oriented shapes, were of poor nutritional quality from high sugar, fat, and sodium amounts [11].

Based on the use of food shapes in marketing toward children, one could assume that offering foods that are shaped to resemble an item a child might be interested in would encourage the consumption of healthy foods, thereby improving the diet quality in children. The Interagency Working Group on Food Marketed to Children, formed by members of the Federal Trade Commission, Centers for Disease Control, Federal Drug Administration and United States Department of Agriculture, found that foods most heavily marketed to children ages 2–17 years were breakfast cereals, snacks foods, and restaurant foods [12]; costing approximately 70% of food marketing expenditures. The commission proposed a guide for marketing to children and urged companies to market foods that are contributing to a healthful diet. Based on the amount of effort companies are expending on finding ways to make foods attractive to children, one might assume that offering children healthy, shaped foods is an efficient venue to beneficially influence children’s eating behaviors.

Studies on the influence of food shape on the intake of these foods are lacking. One study conducted on the effect of different visual characteristics of foods and suggested that visual appeal had a strong influence of consumption of fruit in children ages 4 to 7 years [13]. Another group of researchers examined the influence of shape in respect to size and flavor on liking and wanting of snacks [14] and found that children were more likely to repeatedly eat a healthy food product if it was offered in a smaller size. To date, the results of only one study focus specifically on the effect of offering shaped foods on children’s consumption [15] and there was no significant difference between offering different shaped foods versus foods in their usual shapes. However, the study did not allow children to first acclimate to the food during a run-in period. The present study was designed to improve on previous research on the topic and to evaluate the effect of serving healthy, high-fiber snack foods in normal or shaped form on preschooler’s snack consumption.

## Methods

This study was a cross-over intervention study with repeated exposures to snack foods. The design included one within-subject factor (normal vs. shaped snack). The intervention took place on all 4 days of the week when the child care center was open (the child care center was closed on Fridays) over 9 weeks, with the first three weeks serving as acclimation period for children. The acclimation period had two purposes: 1) children got used to the study foods, so that the consumption of the foods during the data collection period reflected the effect of the food shape, not the novelty of a new type of food or food flavor and 2) children got used to the activities involved in the plate-waste measurement methods. The data was collected from February to March in the spring of 2011.

### Participants and recruitment

We recruited 28 boys and girls attending a local part-time child care center of which 21 completed the study and we included in the analysis. The daycare included half-day sessions in either the morning or the afternoon. The morning and afternoon sessions had two separate classrooms; one for children aged two to three years and one for three to five years. The two morning classes and two afternoon classes formed the four groups of the children in the study.

Recruitment flyers were posted at the child care center and distributed to teachers, administrators, and parents. The inclusion criteria for participation in the study were male and female children aged two to five years old who did not suffer from any food allergies, food restrictions, or digestive diseases, such as Crohn’s Disease or Cystic Fibrosis. All children attending the child care center were invited to participate in this study.

Informed consent was obtained from parents. Parents also completed a questionnaire about their children with questions adapted from the National Health and Nutrition Examination Survey (NHANES). Questions included demographic information as well as health and allergy information, which served as screener for study inclusion. Verbal child assent was obtained from children on each study day. The local Institutional Review Board (IRB) approved of the study.

### Study foods

On the study days, the regularly scheduled snack foods at the child care center were replaced with one of three different high-fiber, low-fat snack foods: banana bread, pancakes, and wrap-type turkey and cheese sandwiches. These foods were developed by the researchers to provide high fiber density (3 to 6 grams per serving) and to be low in total fat. To test the snacks for acceptability, all 3 snacks were field tested in another daycare prior to the onset of this study. Each snack was served to approximately 20 children, who rated the food using an age-appropriate three-level likert scale as either “dislike very much,” “neither like nor dislike,” and “like very much.” Recipes were modified until at least 50% of the children liked the food and at least 80% of the children liked the food or had a neutral response. The snacks were scheduled in a three-week menu rotation in which one of the three snacks was served twice every week and no snack was repeated two days in a row. Table 1 shows the intervention scheme used for the study.

**Table 1:** Intervention scheme for three snacks served in two shapes to preschoolers (n=21)

		**Classroom**							
		**1**		**2**		**3**		**4**	
		**n=3**		**n=7**		**n=8**		**n=3**	
**Intervention in week (1-3 run-in)**	**Snack served**	**Normal**	**Shaped**	**Normal**	**Shaped**	**Normal**	**Shaped**	**Normal**	**Shaped**
4	Pancakes		**X**		**X**	**X**		**X**	
	Banana bread		**X**		**X**	**X**		**X**	
	Sandwiches	**X**		**X**			**X**		**X**
	Pancakes	**X**		**X**			**X**		**X**
5	Pancakes		**X**		**X**	**X**		**X**	
	Banana bread		**X**		**X**	**X**		**X**	
	Sandwiches		**X**		**X**	**X**		**X**	
	Banana bread		**X**		**X**		**X**		**X**
6	Sandwiches	**X**		**X**			**X**		**X**
	Banana bread		**X**		**X**	**X**		**X**	
	Pancakes	**X**		**X**			**X**		**X**
	Sandwiches		**X**		**X**	**X**		**X**	
7	Pancakes	**X**		**X**			**X**		**X**
	Banana bread	**X**		**X**			**X**		**X**
	Sandwiches		**X**		**X**	**X**		**X**	
	Pancakes		**X**		**X**	**X**		**X**	
8	Pancakes	**X**		**X**			**X**		**X**
	Banana bread		**X**		**X**	**X**		**X**	
	Sandwiches		**X**		**X**	**X**		**X**	
	Banana bread	**X**		**X**			**X**		**X**
9	Sandwiches	**X**		**X**			**X**		**X**
	Banana bread	**X**		**X**			**X**		**X**
	Pancakes		**X**		**X**	**X**		**X**	
	Sandwiches	**X**		**X**			**X**		**X**

The snacks were prepared based on standardized recipes and measured to provide energy ranging between 150 and 200 calories per serving of each snack, independent of whether they were served in the normal or shaped form (hands, hearts, or animals). Therefore, banana bread weighed on average 80 grams and provided 170 kilocalories (kcal) in either in the shaped (7 hearts) or normal (2 rectangular slices) form. The pancakes in the shaped (3 hands) and normal (2 round pancakes) form weighed on average 72 grams and provided 150 kcal. The sandwiches in their shaped (3 animals) and normal (2 rectangular sandwich halves) form weighed an average of 95 grams and provided 200 kcal.

All snacks were prepared by the researchers in the local metabolic research kitchen. The software Nutritionist Pro (version 4.05, 2011, Nutrition Coordinating Center: University of Minnesota, St. Paul, MN) was used to analyze the nutritional content of the recipes.

### Procedures

Upon arriving at the daycare, parents completed an interview-based questionnaire about their child’s food intake from that morning. The questionnaire included questions about the time, type, and amount of food consumed. Previous intake was coded as yes (=1) or no (=0). Hunger levels at snack time were not measured directly; however, children’s consumption of food during the meal preceding the study (breakfast or lunch) snacks were recorded and included in the statistical analysis.

Each snack was served in either in shaped or normal form. The randomization scheme ensured that all of the children participating in the study were exposed to both food forms for each of the three snacks on four different occasions. Each child’s food consumption was measured using the plate waste method. In summary, each plate was labeled with the child’s identification number and weighed, the food was plated, and both were weighed together. Following snack time, the plate was returned and any food waste that remained on the plate was weighed and recorded. The total amount of snack consumed was calculated by subtracting the weight of the plate and waste, if any, from the weight of the snack served on the plate.

Standard classroom snack time procedures were followed by the participants and the teachers on test days. Each teacher was instructed to refrain from influencing the eating behavior of the children with verbal or non-verbal communications, such as encouragement to eat, nodding, etc.

### Statistical analysis

**Figure 1:**
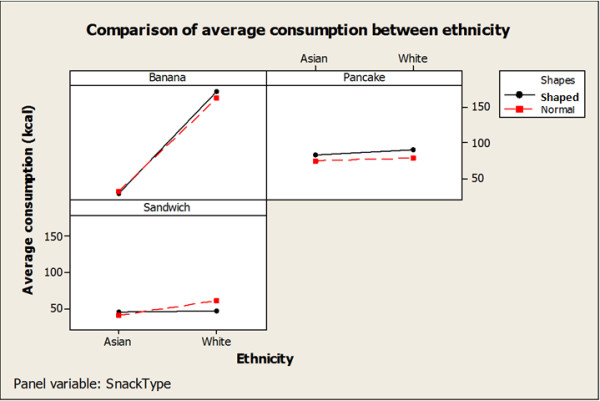
Comparison of kcal means between ethnicities.

Demographic information and plate waste data were entered into Microsoft Office Excel 2010 and snack consumption was calculated. The food consumption prior to the snack (breakfast or lunch) was also entered. SAS (version 9.2, 2008, SAS Institute Inc., Cary, NC) was used for all statistical analyses. Figure 1 was created using Minitab Statistical Software (version 16.1.0, 2010, Minitab Inc., State College, PA).

Snack consumption of all snacks, measured in calories, was analyzed using the General Linear Model procedure and a mixed effects model was used to analyze the data. This analysis included between-subject and with-in subject factors. The between-subject factors were age (older and younger) and race (Asian and Caucasian). The within-subject factors were form (normal and shape) and snack type (banana bread, pancake, and sandwich). Alternative analyses including additional factors such as classroom, age in years, gender did not have an impact on the results. Power analysis showed that only 16 participants would have been needed to show a 65 kcal difference in mean energy consumption between the groups (resulting in 95% power) with statistical significance at p<0.05.

## Results

Twenty-eight subjects were enrolled and twenty-one subjects (75%) completed the protocol in its entirety and were included in the analysis. Five children were withdrawn by their parents due to loss of interest and one child was frequently ill. Based on the participant’s ethnic background, the sample was grouped into Asian or Caucasian children. Only one child did not fit into either ethnic group and was excluded from the analysis (demographics of the participants are in Table 2). Throughout the day, 10 children attended the morning session and 11 the afternoon session. Participation of Asian and Caucasian children was not distributed equally. The number of children participating at each study day and the sample size for each snack observation are reflected in Table 3.

**Table 2:** Sex and race sample characteristics by classroom (n=21)

	**Classroom**				
	**1**	**2**	**3**	**4**	**Total**
Sex					
Boys	1	4	4	2	11 (52%)
Girls	2	3	4	1	10 (48%)
Race					
Asian	0	2	4	2	8 (38%)
Caucasian	3	5	4	1	13 (62%)

**Table 3:** Snack Observations and Days Served

	**Acclimation**		**Intervention**						**Total**	
**Snack**	**Normal (Days)**	**Normal (Observations)**	**Normal (Days)**	**Normal (Observations)**		**Shaped (Days)**	**Shaped (Observations)**		**Total Days Served**	**Total Observations**
				**AM**	**PM**		**AM**	**PM**		
**Pancakes**	2	40	4	33	39	4	37	41	10	190
**Banana Bread**	3	57	4	38	41	4	36	40	11	212
**Sandwiches**	3	59	4	33	41	4	38	42	11	213

*Observation = measured snack food consumption for each child present at study day.

Means and standard deviations for snack consumption by ethnic group are provided in Table 4. Because there were no significant differences in consumption between the different age groups (p = 0.76), age group was not included as a variable in the analyses. There was no significant difference in average snack consumption between the shaped and normal form snacks (p = 0.16).

**Table 4:** Means and standard deviations in kcals of snack foods

**Snack Ethnicity**	**Pancake**		**Banana bread**		**Sandwich**	
	**Normal**	**Shaped**	**Normal**	**Shaped**	**Normal**	**Shaped**
White	79.0 +/- 55.0	88.4 +/- 60.6	161.3 +/- 62.1	168.7 +/- 67.0	58.8 +/- 55.8	48.3 +/- 52.8
Asian	76.6 +/- 46.8	83.6 +/- 59.7	38.1 +/- 54.0	27.8 +/- 46.0	38.7 +/- 24.2	42.1 +/- 32.1

Children who reportedly did not eat breakfast or lunch, ate on average 84.1 calories (kcal) more during the snacks than children whose parents reported that they did eat before the snack (p<0.0001). Figure 1 depicts a comparison of the mean consumption between Caucasian and Asian children. Mean energy intake was significantly higher for Caucasian children compared with Asian children (124.2 versus 59.3 kcal, p<0.01). There was also greater mean consumption of the banana bread by the Caucasian children compared to Asian children (166.2 versus 36.5 kcal, p<0.002). Caucasian children ate significantly more banana bread than pancakes (166.2 versus 82.1 kcal, p<0.02) while no significant difference was observed between the intakes of the pancakes and banana bread in Asian children (36.5 versus 82.0 kcal, p = 0.51).

## Discussion

The aim of this study was to explore a possible method for increasing consumption of foods that supports better diet quality in preschool age children by determining the effect of serving snack foods in normal or shaped form on food consumption. Our study results are in agreement with previous research that showed no difference in consumption between differently shaped snacks [15]. The fact that we found no relationship between snack consumption and shape of snack foods begs the question why so many commercially available foods targeted toward children are frequently in “fun” shapes and packages. One possible explanation is that snack products marketed in the “fun” form may influence what appeals to children in the store but not how much of that food they subsequently eat. Further research in large samples of children and using different foods to explore whether the shape of foods affects purchasing and/or consumption is needed.

The study was designed to explore if children eat more of a healthy food when it is served in a recognizable shape. Results show that there was no effect of snack shape on intake, however, the study results included two unexpected findings: a) results indicate that Asian children ate significantly less than their Caucasian peers and b) Asian children consumed less banana bread. The first finding could be explained by the different consumption patterns between children from various ethnic backgrounds. Xie et al. found that Asian children have overall different nutrient intake patterns compared with Non-Hispanic Caucasian children [16]. However, the possible effect of acculturation and the time frame by which children are acculturated to American eating patterns need to be explored. Ethnicity is strongly associated with the prevalence of overweight and obesity in children [17]. For instance, among 4-year olds, the groups with the lowest prevalence of obesity are non-Hispanic Caucasian and Asians while the highest prevalence is found in American Indian/Native Alaskan, Hispanic, and non-Hispanic Blacks. The factors influencing ethnic differences in childhood obesity are complex combinations of genetics, physiology, culture, socioeconomic status, and environment [18]. In order to better understand the effect of ethnic background on eating patterns, it crucial to understand the underlying mechanisms of these factors [19]. Ethnically distinct eating patterns are well established, for instance, in children ages birth to 11 years old, dietary energy density is significantly lower in Asian children compared to Caucasian children [20]. Asian children have lower intakes of energy, total fat, saturated fat, monounsaturated fats, polyunsaturated fats, and added sugar while Non-Hispanic Caucasian children have higher intakes of saturated fat and lower intakes of fruits [16]. Consideration of familial and cultural eating patterns as well as other social factors is important when developing nutrition interventions.

The strengths of this study included the acclimation period and snack testing prior to the study intervention. The use of the plate-waste procedure, a standardized process for accurately measuring food consumption, resulted high validity of the data. Furthermore, children’s snack consumption accounted for food consumption prior to the test snacks. However, this study also had limitations. The child-care center introduced several new snacks to the menu a month prior to the beginning of the study; this may have influenced the children’s consumption. The number of children participating on each study day was influenced by family’s travel schedules or sick days. Children not participating in the study received the child care center’s snack but were seated with the children participating in the study, thus intake of some of the study participants may have been affected by the social interaction as children would consume snacks based on their peers’ actions. Due to Institutional Review Board instructions we were not allowed to separate participants and non-participants in the classrooms, thus we were not able to control for social facilitation of eating.

## Conclusions

The study was conducted to investigate whether repeated exposure to shaped, healthy, high-fiber snack foods would modify the snack consumption of preschool children, ages 2 to 5 years old. Results indicated no significant differences, however, Caucasian children consumed greater amounts of snack foods than the Asian children did. Children who did not eat during the meal prior to the snack (breakfast or lunch) had increased snack intake. These findings suggest that although food companies spend large amounts of resources on marketing shaped or “fun foods” to children, there might be no association between the shape of foods and children’s consumption of the foods. Future studies conducted in large and diverse samples of children and with long intervention periods are needed to fully evaluate the effect of food shape on children’s food intake.

## Abbreviations

U.S, United States; Kcal, Calories.

## Competing interests

There were no competing interests while completing this study.

## Authors’ contributions

SK and LB designed the study. LB conduced the data collection and manuscript preparation under the guidance of SK. SL and GM analyzed all data. All authors contributed to the interpretation of the data as well as read and approved the final manuscript.

## Authors’ informations

SK led this research study and is associate professor and director of the Coordinated Program in Dietetics in the Department of Nutrition Science at Purdue University. Her research focus area is childhood obesity prevention. LB is an undergraduate honor’s student of SK and will graduate with a Bachelor’s of Science in Dietetics in May 2012. GM is a professor in the Department of Statistics and Associate Dean for Academic Affairs for the College of Science. His research interests include applied statistics, bioinformatics, and statistical computing. SL completed her Masters at Cornell University in Applied Statistics and worked with GM.
